# Utility of Preoperative Cardiac Workup for Children With Very Severe Obstructive Sleep Apnea

**DOI:** 10.1002/lary.70689

**Published:** 2026-06-15

**Authors:** Danny A. Elias, Amber D. Shaffer, Zachary Bennett, Rachel Whelan

**Affiliations:** ^1^ University of Pittsburgh School of Medicine Pittsburgh Pennsylvania USA; ^2^ Department of Otolaryngology University of Pittsburgh Medical Center (UPMC) Children's Hospital of Pittsburgh Pittsburgh Pennsylvania USA

**Keywords:** adenotonsillar hypertrophy, adenotonsillectomy, cardiopulmonary testing, obstructive sleep apnea, pediatric otolaryngology

## Abstract

**Objective:**

We aimed to examine the prevalence and utility of obtaining a preoperative cardiac workup in children with very severe OSA prior to adenotonsillectomy (AT).

**Methods:**

We conducted a review of children 2–18 years with very severe OSA (Apnea‐Hypopnea Index [AHI] ≥ 25) who underwent AT between 2018 and 2024. Wilcoxon rank‐sum and Spearman's rho (*ρ*) tests were performed to determine associations between demographics, cardiac workup, polysomnography parameters, and length of stay [LOS].

**Results:**

Of 144 patients with very severe OSA, 38 patients (26.4%) underwent a preoperative cardiac workup. Three of 38 (7.9%) had abnormal findings; all three were obese, aged 13–16 years, and had preoperative AHI > 50. None underwent additional testing or were started on CPAP preoperatively. There was no association between a cardiac workup, obesity, sex, or age and improvement in AHI. Higher preoperative AHI and younger age correlated to longer LOS (*p* = 0.040, *ρ* = 0.17 and *p* = 0.0007, *ρ* = −0.28, respectively).

**Conclusion:**

In children with very severe OSA, obtaining a preoperative cardiac workup had a low yield of positive findings and was not associated with perioperative course. Adolescent patients with obesity and preoperative AHI > 50 events/h may be at highest risk for abnormal cardiac findings. Our study suggests that a more selective approach to preoperative cardiopulmonary workup may decrease healthcare expenses and minimize delays in care without deleterious effects on patient outcomes and management.

**Level of Evidence:**

3.

## Graphical abstract

In this retrospective study, we aimed to examine the prevalence and utility of obtaining a preoperative cardiac workup in children with very severe obstructive sleep apnea (OSA) prior to adenotonsillectomy. Of 144 patients with very severe OSA, 38 patients (26.4%) underwent a preoperative cardiac workup, and three of those 38 had abnormal findings related to OSA. Overall, we found that, in children with very severe OSA, obtaining a preoperative cardiac workup had a low yield of positive findings and was not associated with the perioperative course.

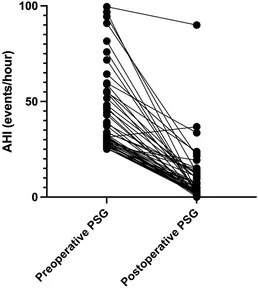

## 1 Introduction

Over the past decade, rates of obstructive sleep apnea (OSA), defined by recurrent airway obstruction leading to desaturation and/or arousals during sleep, have steadily increased in prevalence among children [1, 2]. This increase is due to increased rates of pediatric obesity, improved survival among medically complex children, along with increased OSA awareness and screening through polysomnography [1, 2, 3, 4, 5]. In children, OSA most commonly occurs due to adenotonsillar hypertrophy and adenotonsillectomy (AT) remains an effective first‐line treatment for sleep‐disordered breathing in children, ranging from disruptive snoring to very severe OSA [1, 6, 7].

In adults, chronic, severe, untreated OSA has been associated with negative cardiopulmonary sequelae, including pulmonary hypertension, coronary artery disease, ventricular dysfunction, and heart failure [8, 9]. Pediatric evidence, in contrast, is less robust. Current literature suggests an increased risk of elevated blood pressure in adolescents with OSA as well as possible early cardiac remodeling in untreated pediatric OSA; however, these are smaller studies evaluating patients with any degree of OSA, defined as an apnea‐hypopnea index (AHI) > 2 events per hour, rather than those children with more severe disease [10, 11, 12].

Furthermore, a clearly defined role for dedicated cardiopulmonary testing in children with OSA does not currently exist. Clements and colleagues analyzed the use of cardiopulmonary testing prior to AT in children with severe OSA (AHI ≥ 10 events per hour) and found no association between AHI and abnormal cardiopulmonary findings, suggesting that the decision to conduct cardiopulmonary testing may require considering clinical variables other than OSA severity [6].

While severe OSA (AHI ≥ 10) makes up as much as 14.5% of all pediatric OSA cases, Clements and colleagues further subdivided severe OSA into AHI ranges and found that 67.9% of those with severe OSA had an AHI between 10 and 29.9 events per hour [6, 13]. There is currently no consensus on an AHI cut point for very severe OSA in children; studies have used values ranging from 19 to 30 events per hour [14, 15]. Based on expert consensus, we chose to utilize 25 events per hour as our definition of very severe disease.

To date, the long‐term cardiovascular impact of severe OSA in children and evidence‐based guidelines for preoperative cardiac testing are unclear. As a result, children found to have very severe OSA oftentimes undergo preoperative cardiopulmonary testing based largely on adult evidence [10, 12]. In this study, we sought to evaluate the clinical utility of preoperative cardiopulmonary testing in children with very severe OSA (which we defined as an AHI ≥ 25 events per hour) prior to undergoing AT. Specifically, we aimed to determine the frequency of abnormal findings on cardiac testing, assess whether these findings were associated with changes in perioperative management or outcomes, and identify factors associated with the decision to obtain preoperative cardiopulmonary evaluation.

## 2 Materials and Methods

### 2.1 Study Design/Data Collection

This study was a retrospective chart review of patients aged 2–18 years undergoing AT for very severe OSA, defined as a total AHI ≥ 25 events per hour on preoperative overnight polysomnography (PSG) at a single‐site tertiary children's hospital between 2018 and 2024. All PSGs were performed overnight in a sleep laboratory and were attended by a laboratory technician. PSG data were interpreted by a physician specializing in sleep medicine. The Institutional Review Board at the University of Pittsburgh School of Medicine approved this study (IRB# STUDY23050199). Patients with the most recent preoperative PSG showing AHI < 25 were excluded.

All data were collected from electronic medical records. Demographic and patient data included age at time of surgery, race, sex, most recent height, weight, body mass index (BMI), sex‐specific BMI‐for‐age percentile, and co‐morbidities (obesity, diabetes, genetic abnormalities, craniofacial malformations, asthma, premature birth, bronchopulmonary dysplasia, hypertension, heart disease, and seizure disorders). Sleep study parameters included study date, study type (diagnostic study versus split night study), total AHI, obstructive apnea‐hypopnea index (OAHI), central apnea index (CAI), sleep efficiency, sleep latency, and oxygen nadir. Total AHI was calculated as the total number of apneas and hypopneas detected on PSG per hour. CAI was calculated as the sum of apneas and hypopneas showing no respiratory effort on PSG per hour. OAHI was calculated as the sum of apneas and hypopneas showing continued respiratory effort on PSG per hour. Operative data included tonsil size, adenoid percent occlusion of the nasopharynx, and surgical complication information from the intraoperative report. Postoperative data included oxygen nadir, need for supplemental oxygen (i.e., face mask, nasal cannula, reintubation), pediatric intensive care unit (PICU) versus floor admission, length of stay (LOS), and data from the first postoperative PSG (when performed). Postoperative PSG analysis was limited to total AHI, OAHI, and CAI.

We then recorded data relevant to any cardiac workup obtained as new preoperative testing, including chest radiograph, electrocardiogram (ECG), and/or echocardiogram. Workups obtained as routine testing for those with preexisting cardiopulmonary conditions or for any reason other than severe PSG findings and preoperative optimization were not included in the analysis. Any relevant positive findings were defined as those that could be linked to cardiopulmonary sequelae of untreated OSA, including right heart strain, ventricular hypertrophy, and/or diastolic dysfunction. Congenital structural findings were recorded but not considered positive findings. To account for individual practice variability in ordering cardiac workup, we additionally noted the provider who evaluated each patient after their PSG, given this is when the decision to order a cardiac workup or not is made. For patients with positive findings, preoperative anesthesia and otolaryngology documentation were reviewed for cardiopulmonary symptoms and presence of special management considerations.

### 2.2 Statistical Analysis

Continuous variables were treated as nonparametric. Associations between binary independent variables versus LOS and change in AHI were evaluated using Wilcoxon rank‐sum testing. Continuous independent variables' associations were evaluated using Spearman's rho (*ρ*) tests. Time interval between preoperative PSG and cardiac workup was calculated as the number of days between the PSG and the date of the latest cardiac workup (chest radiograph, ECG, or echocardiogram).

To identify providers with significantly higher ordering frequencies, exact logistic regression was performed on the rates of ordering cardiac workup for each provider. After dividing the cohort by provider tendency (high‐rate of cardiac workup versus standard‐rate), we then performed Student's *t*‐tests on the AHIs of those who received a cardiac workup versus those who did not for each group.

Wilcoxon rank‐sum tests were conducted to compare age at surgery, sex‐specific BMI‐for‐age percentile, total AHI, and LOS between those who received a cardiac workup and those who did not. Additional PSG parameters were summarized descriptively and were not included in inferential analyses. Categorical variables obesity status, Trisomy 21, craniofacial abnormalities, congenital heart disease, and presence of genetic conditions other than Trisomy 21 were compared between groups using Fisher's exact testing. A multivariable logistic regression model including age at surgery, sex‐specific BMI‐for‐age percentile, and total AHI was then constructed to assess their independent associations with receipt of cardiac workup, and odds ratios (OR) with confidence intervals (CI) were also calculated. Categorical comorbid variables were not included in the model due to low prevalence and limited statistical power. Fisher's exact testing was also used to compare the postoperative variables of supplemental oxygen requirement, planned (scheduled) PICU admission, and unplanned (nonscheduled) PICU admission between cardiac workup and no cardiac workup groups.

Due to the small number of patients with abnormal findings on cardiac workup, the data from these patients were evaluated qualitatively.

## 3 Results

### 3.1 Clinical Characteristics

One hundred forty‐four patients met inclusion criteria, 95 (66%) were male. Patient demographic and clinical data are summarized in Table 1. Median age at surgery was 5.81 years. 63.9% of patients identified as white, and 25.7% as black. Median sex‐specific BMI‐for‐age percentile was 96.99 (range 0–100), and 38.9% of patients carried a diagnosis of obesity in their record. Following obesity, congenital heart disease (13.9%), asthma (11.8%), craniofacial malformations (11.1%), and premature birth (11.1%) were the most common comorbidities. Three patients (2.1%) were on positive pressure therapy preoperatively: two on continuous positive airway pressure (CPAP), one on bilevel positive airway pressure (BPAP).

**TABLE 1:** Demographics and comorbidities of pediatric patients aged 2–18 years with AHI ≥ 25 treated with adenotonsillectomy.

	Overall, *n* = 144 no. (%)
Sex	
Male	95 (66.0%)
Female	49 (34.0%)
Race	
White	92 (63.9%)
Black	37 (25.7%)
Multiple	4 (2.8%)
Other	2 (1.4%)
Not specified	9 (6.3%)
Age at surgery, years	5.81 (2.01–17.98)^a^
Sex‐specific BMI‐for‐age percentile	96.99 (0–100)^a^
Comorbidities	
Obesity	56 (38.9%)
Congenital heart disease	20 (13.9%)
Asthma	17 (11.8%)
Craniofacial malformations	16 (11.1%)
Prematurity	16 (11.1%)
Other genetic abnormality	12 (8.3%)
Trisomy 21	12 (8.3%)
Neuromuscular disorder	8 (5.6%)
Epilepsy/siezure disorder	5 (3.5%)
Pulmonary hypertension	5 (3.5%)
Systemic hypertension	5 (3.5%)
Diabetes	4 (2.8%)
Bronchopulmonary dysplasia	2 (1.4%)
No comorbidities	51 (35.4%)

^a^ Median (range).

### 3.2 Preoperative Studies and Testing

Table 2 summarizes preoperative PSG data in all patients, 94.4% of which underwent a diagnostic baseline study without PAP titration. Median AHI was 35.4 events/h (range 25.0–118.1); median OAHI was 32.3 events/h (range 12.9–117.5, *n* = 136); median oxygen nadir was 75% (range 31–91).

**TABLE 2:** Preoperative polysomnography results of pediatric patients aged 2–18 years with AHI ≥ 25 treated with adenotonsillectomy by receipt of preoperative cardiopulmonary testing.

	Overall, *n* = 144 no. (%)	No cardiopulmonary testing, *n* = 106 no. (%)	Cardiopulmonary testing, *n* = 38 no. (%)	*p*
Preoperative PSG type				
No titration (baseline study)	136 (94.4%)	100 (94.3%)	36 (94.7%)	
Split night (PAP titration)	8 (5.6%)	6 (5.7%)	2 (5.3%)	
Preoperative PSG results				
AHI, events/h	35.4 (25–118.1)^a^	34.0 (25–99.6)^a^	51.8 (25–118.1)^a^	0.013
OAHI, events/h	32.3 (12.9–117.5)^a^	31.4 (12.9–94.6)^a^	40.3 (23.6–117.5)^a^	
CAI, events/h	1.1 (0–50.7)^a^	1.1 (0–50.7)^a^	0.8 (0–34.7)^a^	
Sleep efficiency, %	83.1 (22.5–99.1)^a^	83.4 (22.5–99.1)^a^	81.2 (41.2–97.8)^a^	
Sleep latency, minutes	20.8 (0–241.1)^a^	22.8 (0–241.1)^a^	9.5 (0–172)^a^	
Oxygen saturation nadir, %	75 (31–91)^a^	76 (31–91)^a^	71.5 (31–89)^a^	

^a^ Median (range).

Thirty‐eight patients (26.4%) underwent dedicated preoperative cardiac workup. Thirty‐one patients (21.5%) had a chest X‐ray, 27 (18.8%) had an ECG, and 29 (20.1%) had an echocardiogram (Table 3). Median number of days from preoperative PSG to cardiac workup was 68.5 (range 10–546). Univariate testing and multivariate logistic regression showed total AHI to be independently associated with receipt of preoperative cardiac workup (OR 1.02, 95% CI 1.01–1.04, *p* = 0.013). Age at surgery and sex‐specific BMI‐for‐age percentile were not associated with receipt of cardiac workup (*p* = 0.96 and *p* = 0.89, respectively). None of categorical variables including obesity status, Trisomy 21, craniofacial abnormalities, congenital heart disease, or presence of other genetic conditions were associated with receiving a cardiac workup (no significant difference in the proportions of these variables between groups; all *p* values > 0.05) (Table 4).

**TABLE 3:** Rates of preoperative cardiopulmonary testing in pediatric patients with very severe OSA.

	Overall, *n* = 144 no. (%)
Cardiopulmonary workup	38 (26.4%)
Chest X‐ray	31 (21.5%)
Echocardiogram	29 (20.1%)
Electrocardiogram	27 (18.8%)
Abnormal findings on workup	3 (7.9%)^a^

^a^ Of the *n* = 38 with a cardiopulmonary workup.

**TABLE 4:** Demographics and comorbidities in pediatric patients with very severe OSA by receipt of preoperative cardiopulmonary testing.

	No cardiopulmonary testing, *n* = 106 no. (%)	Cardiopulmonary testing, *n* = 38 no. (%)	*p*
Demographics			
Age at surgery, years	5.75 (2.01–18.0)^a^	6.27 (2.01–17.91)^a^	0.96
Sex‐specific BMI‐for‐age percentile	96.9 (0–100)^a^	98.0 (0.24–100)^a^	0.89
Comorbidities			
Obesity	39 (36.8%)	17 (44.7%)	0.44
Down syndrome	10 (9.4%)	2 (5.3%)	0.73
Craniofacial abnormalities	12 (11.3%)	4 (10.5%)	1
Congenital heart disease	18 (17.0%)	2 (5.3%)	0.10
Other genetic abnormalities	9 (8.5%)	3 (7.9%)	1

^a^ Median (range).

Of the patients with a cardiac workup, only three (7.9%) had an abnormal finding potentially related to OSA (Table 3). These findings included a mildly hypertrophied interventricular septum on echocardiogram (*n* = 1, Patient A), abnormal right ventricle diastolic function on echocardiogram (*n* = 1, Patient B), and right ventricle hypertrophy via low voltage QRS complex on ECG (*n* = 1, Patient C). All three patients with abnormal findings were obese (sex‐specific BMI‐for‐age percentile > 99th percentile) and had preoperative AHI > 50 events per hour. Patient A and Patient C were both male and age 13 years at the time of their respective surgeries. Patient B was female and age 16 years at the time of her surgery. None were placed on CPAP or BPAP preoperatively. None had cardiopulmonary symptoms outside of those attributable to OSA. Table 5 details demographics, PSG results, and comorbidities for these three patients.

**TABLE 5:** Demographics, PSG characteristics, and comorbidities in three patients with abnormal findings on preoperative cardiopulmonary testing.

	Patient A	Patient B	Patient C
Demographics			
Gender	Male	Female	Male
Age at surgery, years	13.2	16.2	13.7
Sex‐specific BMI‐for‐age percentile	99.9	99.4	99.8
Preoperative PSG results			
AHI, events/h	83.7	59.6	82.0
OAHI, events/h	81.2	24.9	81.5
CAI, events/h	2.5	34.7	0.5
Sleep efficiency, %	41.2	78.2	76.7
Sleep latency, minutes	2.8	5.7	4.7
Oxygen saturation nadir, %	79	70	65
Comorbidities^a^			
Obesity	Yes	Yes	Yes
Systemic hypertension	Yes	No	No
Fragile X syndrome	No	No	Yes

^a^ Comorbidities not listed here were not present in any of the above patients.

Given current lack of consensus, we also examined the decision to obtain a preoperative cardiac workup. In our cohort, 21 different providers evaluated patients after their PSG, made up of both physicians and advanced practice providers. Of the 21, two (9.5%) ordered significantly more cardiac workups than the rest of the providers (*p* = 0.03 and *p* = 0.02), accounting for 16 of the 38 total workups (42.1%). These providers were both pediatric otolaryngologists. While there were no significant associations between undergoing a preoperative cardiac workup, obesity, sex, preoperative OAHI, or preoperative oxygen nadir and LOS when looking at the entire cohort in aggregate, a sensitivity analysis excluding these providers showed that those who received a cardiac workup had a significantly higher AHI than those who did not (55.7 events per hour versus 41.9 events per hour, *p* = 0.04).

### 3.3 Postoperative Course

Table 6 details the postoperative data for the study cohort. Median LOS was 1 day (range 1–11), and 74 patients (51.4%) had a scheduled PICU stay. Four patients (2.8%) were transferred from a nursing floor to the PICU. Thirty‐seven patients (26.4%) required supplemental oxygen postoperatively. Three patients (2.1%) were on CPAP or BPAP during their hospital stay, two of whom were continued on PAP after discharge. One patient remained intubated until postoperative day five due to suspected pneumonia. No patients developed postobstructive pulmonary edema.

**TABLE 6:** Postoperative characteristics in pediatric patients with very severe OSA by receipt of preoperative cardiopulmonary testing.

	Overall, *n* = 144 no. (%)	No cardiopulmonary testing, *n* = 106 no. (%)	Cardiopulmonary testing, *n* = 38 no. (%)	*p*
Hospital course				
Length of stay, days	1 (1–11)^a^	1 (1–7)^a^	1 (1–11)^a^	0.86
Facemask/nasal cannula/blowby oxygen	36 (25.0%)	25 (23.6%)	11 (28.9%)	
CPAP/BPAP during stay	3 (2.1%)	1 (0.9%)	2 (5.3%)	
Prolonged postoperative intubation	1 (0.7%)	0 (0%)	1 (2.6%)	
Any supplemental oxygen (any combination of the above three)	37 (25.7%)	25 (23.6%)	12 (31.6%)	0.39
Location of hospital course				
Planned PICU stay	74 (51.4%)	51 (48.1%)	23 (60.5%)	0.26
Non‐PICU/nursing floor	66 (45.8%)	51 (48.1%)	15 (39.5%)	
Unplanned PICU stay	4 (2.8%)	4 (3.8%)	0 (0%)	0.57
Postoperative PSG data				
Number of patients	50 (34.7%)	42 (39.6%)	8 (21.1%)	
AHI	3.6 (0–90)^a^	3.1 (0–90)^a^	6.0 (1.1–21.5)^a^	
OAHI	2.3 (0–90)^a^	2 (0–90)^a^	4.7 (0–21.5)^a^	
ΔAHI	26.8 (−5.5–94)^a^	26.4 (−5.5–94)^a^	35.7 (23.3–81.4)^a^	
CPAP/BPAP posthospital stay	12 (8.3%)	9 (8.5%)	3 (7.9%)	

^a^ Median (range).

Of the 38 patients who underwent preoperative cardiac workup, median LOS was 1 day (range 1–11) and 23 (60.5%) had a scheduled PICU stay. None of these patients had a nonscheduled PICU stay. Twelve patients (31.6%) required supplemental oxygen postoperatively. There were no significant differences between median LOS or rates of any of the above postoperative characteristics and receiving a cardiac workup (Table 6). Postoperative PSG data were not compared between those who received a cardiac workup and those who didn't due to the low number of postoperative PSGs conducted (see below).

Among the three patients with abnormal findings on preoperative cardiac workup, all were discharged on postoperative day one with zero intraoperative complications listed on the operative report. Two of the three preoperative anesthesia reports noted the abnormal cardiac findings; none mentioned any changes or special considerations in their intraoperative planning documentation. No patients had dedicated cardiac anesthesiology for their surgery. Preoperative otolaryngology documentation similarly made no mention of changes to the routine operative plan. One of the three patients required supplemental oxygen (nasal cannula) postoperatively. None were started on CPAP or BPAP postoperatively.

A higher preoperative AHI was associated with longer LOS, with a weakly positive correlation (*p* = 0.040, *ρ* = 0.17). Younger age was also associated with a longer LOS, with a weak‐moderate relationship (*p* = 0.0007, *ρ* = −0.28).

**FIGURE 1:**
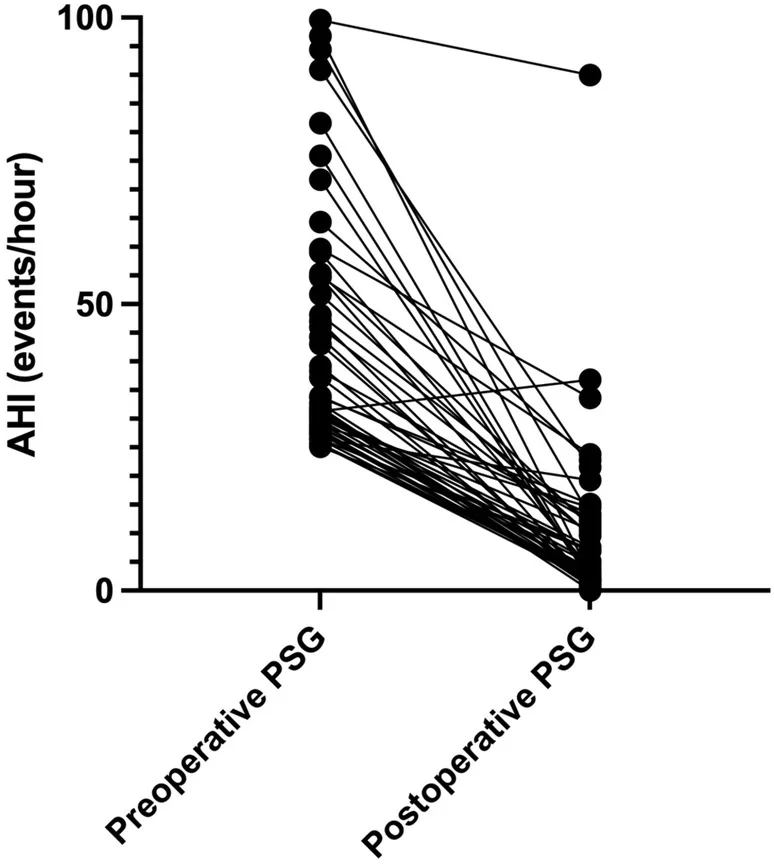
Pre‐ versus postadenotonsillectomy AHI (*n* = 50) (AHI: Apnea‐hypopnea index; PSG: Polysomnogram).

Fifty patients (34.7%) underwent postoperative PSG (Table 6). Among these 50, median AHI was 3.6 events per hour (range 0–90), median OAHI was 2.3 events per hour (range 0–90), and median CAI was 0.6 events per hour (range 0–7.6). Compared to baseline severity, the median improvement in AHI was 26.8 events per hour (range 5.5–94); one patient's AHI increased postoperatively (Figure 1).

## 4 Discussion

### 4.1 Utility of Cardiopulmonary Testing

Our work contributes to the growing body of evidence that postoperative complications in children with very severe OSA are rare and that no differences in operative or postoperative planning were observed in those who received preoperative cardiac workup. It is also among the first to examine predictors associated with receiving a cardiac workup in the context of provider‐dependent decision‐making.

Among 144 children undergoing AT for very severe OSA, 38 patients underwent a preoperative cardiac workup with only three abnormal findings elucidated. Having a positive cardiac finding was not associated with changes in perioperative workup/treatment, hospital length of stay, or complication profile. Furthermore, undergoing cardiac workup was not associated with differences in postoperative course, with no significant differences in length of stay, postoperative oxygen requirements, or PICU admission between those who received the workup and those who did not. The similar rates of PICU admission and median LOS between the two groups suggest that forces other than cardiac workup may be influencing the decisions around PICU admission and discharge date. Notably, despite higher preoperative AHI among patients who underwent cardiac workup, postoperative outcomes were similar, suggesting that disease severity did not translate into increased perioperative risk in this cohort.

Our work also found AHI to be an important factor for ordering a cardiac workup, with higher AHI being associated with increased odds of undergoing cardiac workup (OR 1.02). Although this OR is modest, the wide range of observed AHI values suggests that differences in OSA severity may meaningfully influence the clinical decision making behind ordering cardiac workup. Provider‐level variation was also observed, with two otolaryngologists accounting for a disproportionate number of cardiac workups. While excluding these providers did not lead to a meaningful difference in the relationship between AHI and receipt of a cardiac workup, this variability likely reflects the absence of standardized guidelines for ordering such studies in very severe pediatric OSA.

The existing body of literature examining the utility of cardiopulmonary workup for patients with very severe OSA demonstrates mixed findings and thus provides mixed recommendations. Amin and colleagues found that in children aged 2–18 years, higher AHI correlated positively with right ventricle dimension, suggesting a potential risk of right heart remodeling in patients with more severe OSA [16]. Clements and colleagues found no significant association between OAHI and abnormalities on cardiopulmonary testing in patients with OAHI greater than 30 [6]. These studies suggest that while severe OSA in children may have some effect on cardiac morphology, this does not appear to translate to a clinical impact based upon cardiac testing results. This is perhaps further supported by previous work from Beyazal, who showed that children aged 8–18 years who were dominant athletes had evidence of statistically significant cardiac remodeling when compared to a beginner‐athlete cohort, yet demonstrated no difference in ejection fraction [17]. When compared to the adverse cardiopulmonary effects OSA has on adult patients, this notion speaks to the generally higher physiologic resilience and plasticity exhibited by children under various biological insults versus adults (such as in high‐intensity exercise or COVID‐19 infection) [18, 19].

In a study by Katz and colleagues, it was found that comorbidities including craniofacial or airway anomalies and prematurity were the strongest predictors of postoperative respiratory complications in children aged 0–18 years undergoing AT [20]. Interestingly, Katz and colleagues found that while younger age also increased the odds of complications (< 6 years), obesity was not a statistically significant predictor of complications. While our findings similarly did not demonstrate an association between obesity and perioperative outcomes, this contrasts with several other studies that have identified obesity as a risk factor for increased perioperative respiratory complications in children undergoing AT. For instance, in their cohort of over 20,000 children, Lavin and Shah found that 16.2% of obese patients undergoing AT had postoperative respiratory complications, including minor events like hypoxemia and major events like acute respiratory failure, compared to only 9.6% of nonobese patients [21]. Lee and colleagues similarly found higher BMI z‐score to be independently associated with overnight airway depression [22]. One possible explanation for this discrepancy is the relatively high baseline prevalence of elevated sex‐specific BMI‐for‐age percentile in our cohort, combined with a relatively small overall sample size, which may limit the ability to detect differences between obese and non‐obese patients. Another possibility may be differences in how postoperative complications are defined. In the present study, we defined complications as requiring unplanned PICU admission, prolonged intubation, reintubation, or the development of post‐obstructive pulmonary edema, while others may have defined minor events such as temporary desaturations as a complication.

In our study, the three patients with abnormal cardiopulmonary findings related to OSA had a shared demographic and clinical history: obese adolescents with AHI > 50 events per hour on PSG. This suggests that in patients with these risk factors, obtaining preoperative cardiopulmonary testing could have a higher yield. However, it is important to note that perioperative complication profiles were not different between groups. Clinically, these findings suggest that while cardiopulmonary testing may identify abnormalities in select patients, it may not meaningfully alter perioperative management or postoperative outcomes in most cases. Thus, while obese adolescents with very severe OSA appear to be at higher risk to have cardiopulmonary findings on testing, younger, otherwise medically complex children are likely at higher risk for perioperative complications. Overall, a more individualized approach to preoperative evaluation may be warranted, particularly in the absence of standardized guidelines.

### 4.2 Limitations

Given the majority of children with OSA have borderline, mild, or moderate OSA, examining patients with very severe OSA inherently yields a relatively small sample size [13]. Further, only a minority of this cohort underwent cardiopulmonary workup, thus limiting our ability to detect statistically significant associations.

As is likely the case in larger, academic centers that tend to manage the majority of severe OSA in pediatric patients, individual provider practices within a large group vary and thus the decision/threshold to obtain a cardiopulmonary workup also varies by provider, limiting this work's ability to interpret the reasoning behind ordering such a workup. This limits the generalizability of the present findings to other settings. Similarly, provider preference also impacts which patients were sent to the PICU postoperatively, with potential effects on postoperative length of stay, again limiting generalizability.

Additionally, without directly approaching the anesthesiologists or otolaryngologists who managed our cohort of patients, it is impossible to know with absolute certainty whether special consideration was given when patients had received cardiac workups preoperatively. Future studies should consider a prospective review to have an opportunity to approach providers directly.

Lastly, the retrospective nature of the study introduces inherent limitations including selection bias, low incidence rates of variables like postoperative PSGs, and the inability to standardize study groups.

## 5 Conclusion

Our study suggests that obtaining a preoperative cardiopulmonary workup in children with very severe OSA and indications for an AT has a low yield and was not associated with differences in perioperative course even when abnormalities were discovered. Patients at higher risk for cardiopulmonary abnormalities appear to be adolescents with obesity and baseline AHI > 50 events/h. Thus, this subgroup may have a higher likelihood of abnormal findings on preoperative chest X‐ray, ECG, and/or echocardiography. While further prospective investigation is warranted to validate these findings in a larger, standardized cohort as well as further elucidate individual provider reasoning for specific preoperative testing, a more selective approach to preoperative cardiopulmonary workup may decrease healthcare expenses and minimize delays in care without deleterious effects on patient outcomes and management.

## Funding

The authors have nothing to report.

## Conflicts of Interest

The authors declare no conflicts of interest.

## Data Availability

The data that support the findings of this study are available on request from the corresponding author. The data are not publicly available due to privacy or ethical restrictions.
